# Personalized Embryo Transfer Improves Live Birth Rates in Recurrent Implantation Failure: A Propensity Score‐Matched Prospective Cohort Study With Window of Implantation Stability Analysis

**DOI:** 10.1002/rmb2.70081

**Published:** 2026-07-22

**Authors:** Yasuhiro Ohara, Kurumi Abe, Saki Saito, Aya Karino, Masakazu Doshida, Takumi Takeuchi, Hidehiko Matsubayashi, Tomomoto Ishikawa, Mika Handa, Tsuyoshi Takiuchi, Michiko Kodama

**Affiliations:** ^1^ Department of Reproductive Medicine Reproduction Clinic Osaka Osaka Japan; ^2^ Department of Obstetrics and Gynecology, Graduate School of Medicine Osaka University Suita city Osaka Japan; ^3^ Department of Reproductive Medicine Reproduction Clinic Tokyo Tokyo Japan; ^4^ Department of Clinical Genomics, Graduate School of Medicine Osaka University Suita city Osaka Japan

**Keywords:** endometrial receptivity test, personalized embryo transfer, real‐time quantitative polymerase chain reaction, recurrent implantation failure, window of implantation stability

## Abstract

**Purpose:**

To evaluate the clinical efficacy of ERPeak^SM^‐guided personalized embryo transfer (pET) in recurrent implantation failure (RIF) and non‐RIF patients, and assess the temporal stability of the window of implantation (WOI).

**Methods:**

This prospective cohort study included patients undergoing frozen embryo transfer (FET) with good‐quality embryos. In hormone replacement therapy (HRT) cycles, RIF patients received pET (*n* = 199) or non‐personalized ET (npET, *n* = 272); non‐RIF patients received pET (*n* = 74) or npET (*n* = 351). Propensity score matching (PSM) adjusted for confounders. modified Natural cycle (mNC) FET was analyzed using inverse probability of treatment weighting (IPTW). WOI stability was evaluated via Kaplan–Meier analysis.

**Results:**

Following PSM in RIF patients, pET significantly improved live birth rates (LBR) compared to npET (41.0% vs. 23.6%, *p* < 0.001) and reduced miscarriage rates (17.4% vs. 36.2%, *p* = 0.014). In non‐RIF patients, pET did not improve LBR (35.8% vs. 25.4%, *p* = 0.26). IPTW‐adjusted mNC‐FET analysis showed significantly higher LBR in the pET group (46.3% vs. 19.2%, *p* = 0.026). Kaplan–Meier analysis revealed WOI stability declines significantly faster in patients ≥ 38 years (median 30.0 months, *p* = 0.0059).

**Conclusions:**

ERPeak^SM^‐guided pET significantly improves LBR in RIF patients undergoing HRT and mNC‐FET, but lacks benefit in non‐RIF patients. WOI is dynamic; reevaluation is recommended for older patients.

## Graphical abstract

ERPeak^SM^‐guided embryo transfer doubled live birth rates in recurrent implantation failure patients but showed no benefit in non‐RIF patients. Window of implantation destabilizes faster in women ≥ 38 years (median 30 months vs. not reached, *p* = 0.0059), warranting earlier reevaluation.

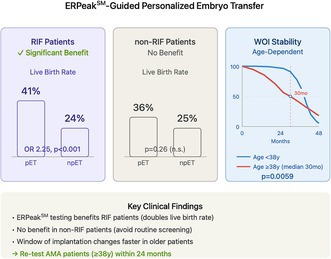

## 1 Introduction

Recurrent implantation failure (RIF) remains one of the most challenging obstacles in assisted reproductive technology (ART). Successful implantation is a highly coordinated process that strictly depends on the synchronization between a competent embryo and a receptive endometrium [1, 2]. The period during which the endometrium is conducive to embryo attachment and invasion is known as the window of implantation (WOI). While standard hormone replacement therapy (HRT) protocols assume a uniform WOI across all patients, accumulating evidence suggests that a displaced WOI—where the endometrium is either pre‐receptive or post‐receptive at the time of standard transfer—is a significant contributing factor to RIF [3].

To address this asynchrony, various molecular diagnostic tools have been developed to assess endometrial receptivity at the genetic level, allowing for personalized embryo transfer (pET) [4, 5, 6, 7]. These tests aim to pinpoint the exact timing of the WOI, thereby optimizing the synchronization between the embryo and the endometrium. However, the clinical utility of these gene expression assays remains a subject of intense debate. While several observational studies and randomized controlled trials (RCTs) have reported that pET significantly improves clinical outcomes in patients with RIF [8, 9], other robust RCTs and meta‐analyses have failed to demonstrate a clear benefit of pET over standard timing, particularly in unselected patient populations or those undergoing their first IVF cycle [10, 11, 12, 13, 14, 15].

This discrepancy in the literature highlights a critical gap in our understanding of the specific patient populations that truly benefit from endometrial receptivity testing. It remains unclear whether the lack of efficacy observed in some studies is due to the inclusion of non‐RIF patients, variations in the testing platforms, or the inherent temporal instability of the WOI itself. Furthermore, while gene expression testing is predominantly utilized in HRT cycles, its applicability and efficacy in modified natural cycle frozen embryo transfer (mNC‐FET) have not been thoroughly investigated.

Therefore, this prospective cohort study was designed to comprehensively evaluate the clinical impact of pET guided by a real‐time quantitative polymerase chain reaction (RT‐qPCR)‐based endometrial receptivity test (ERPeak^SM^). Specifically, we aimed to: (1) compare the live birth rates (LBR) and miscarriage rates (MR) between pET and non‐personalized ET (npET) in both RIF and non‐RIF populations to clarify the clinical indications for testing; (2) explore the utility of ERPeak^SM^‐guided transfer in mNC‐FET through an inverse probability of treatment weighting (IPTW) sub‐analysis; and (3) assess the temporal stability of the WOI over time using Kaplan–Meier survival analysis to establish evidence‐based retesting guidelines.

## 2 Materials and Methods

### 2.1 Patient Characteristics

This prospective cohort study examined obstetric outcomes from Japanese infertility patients (*N* = 1,295, age range: 20–53 years, median: 40 years) of all ages who received in vitro fertilization (IVF) at a private Japanese fertility clinic between August 2022 and December 2023. RIF was defined as the failure to establish a clinical pregnancy following a minimum of three consecutive embryo transfers, each involving the transfer of one or two morphologically high‐grade blastocysts (Gardner score ≥ 3BB on day 5) [16]. In Japan, ERPeak^SM^ testing is currently being evaluated under the Advanced Medical Care framework. Under this system, testing for non‐RIF patients is permitted for those of advanced maternal age (≥ 35 years) or who are planning to transfer highly valuable embryos (e.g., euploid embryos). Patients in the non‐RIF group who opted for testing were thoroughly counseled regarding the lack of established evidence for routine use, and testing was performed based on shared decision‐making.

Prior to enrollment, all participants underwent a standardized infertility workup comprising transvaginal ultrasound, hysteroscopy, and endometrial biopsy to screen for chronic endometritis. Any identified intrauterine pathology—including hydrosalpinx, endometrial polyps, submucosal fibroids, and chronic endometritis—was corrected before the study period. Systemic conditions including thyroid disorders and thrombophilia were also evaluated and managed accordingly. Patient data were extracted from the institutional electronic medical records.

### 2.2 Endometrial Preparation

For HRT cycles, endometrial preparation was initiated in both the mock cycle (pET group only) and the actual transfer cycle (all patients). Oral or transdermal estradiol (Premarin; Pfizer, transdermal patch, or a combination as clinically indicated) was commenced on day 4 (spontaneous or induced menstruation). Once transvaginal sonography confirmed a trilaminar endometrial pattern with a thickness of at least 7.0 mm, typically within two weeks of menstrual onset, progesterone supplementation was initiated using a combination of vaginal progesterone capsules (Utrogestan; Fuji Pharma 400 mg twice daily, total 800 mg/day) and oral chlormadinone acetate (Lutoral; Fuji Pharma 2 mg three times daily). The first day of progesterone administration was designated as “*P* + 0.” Serum progesterone was routinely measured on the day of embryo transfer, and patients with levels below 10 ng/mL received additional supplementation.

In the modified natural ovulation cycle (mNC), letrozole (Femara; Novartis, Japan) 2.5 mg was taken orally for two days starting on the third day of menstruation. Around the tenth day of menstruation, when the endometrial thickness was 7.0 mm or more and the maximum follicle diameter exceeded 16 mm, 10,000 units of human chorionic gonadotropin (HCG Mochida; Mochida Pharmaceutical Co, Japan) were administered subcutaneously 159 ± 1 h before embryo transfer. To supplement progesterone, Lutoral tablet 2 mg was taken three times daily starting from the day of ovulation confirmation.

### 2.3 Endometrial Biopsy

Patients who opted for pET underwent a dedicated mock cycle, conducted a median of 2.0 months (interquartile range: 1.0–3.0 months) before their actual embryo transfer cycle. In the HRT mock cycle, endometrial sampling was performed on day *P* + 5 (corresponding to 113 h after the initiation of progesterone) using the ENDOSUCTION aspiration catheter (Hakko Company Ltd.; Nagano, Japan), which was advanced to the uterine fundus. The aspirated tissue was immediately immersed in a cryotube prefilled with RNAlater RNA stabilization reagent (Invitrogen, Waltham, MA, USA) and mixed by inversion ten times. The sample was then maintained at 4°C for a minimum of four hours before being dispatched to the testing laboratory at ambient temperature (15°C–25°C) for gene expression analysis. In the mNC, a biopsy was performed 7 days (159 ± 1 h) after hCG administration using the same procedure as in HRT.

### 2.4 Endometrial Receptivity Testing

Endometrial receptivity testing was performed using ERPeak^SM^ (CooperSurgical Inc.; Livingston, NJ, USA), a 48‐gene RT‐qPCR assay. The test development and validation have been previously described by our group [5, 17]. Briefly, 48 genes were selected from 184 candidate genes through discriminant functional analysis, explaining > 99.5% of total variance in endometrial receptivity status. The assay classifies the endometrium as Receptive (±0, optimal timing), Pre‐receptive (+1, endometrium not yet ready, requiring 24‐h delay in embryo transfer), or Post‐receptive (−1, endometrium past optimal receptivity, requiring 24‐h advancement in transfer). The assay was validated in 173 independent samples, demonstrating robust accuracy (CooperSurgical, internal data).

### 2.5 Embryo Transfer

In the npET group, embryo transfer was scheduled at the standard timing of *P* + 5 in HRT cycles, or five days following confirmed ovulation in mNC cycles. In the pET group, the transfer timing was individualized based on the ERPeak^SM^ test result, with the final decision to proceed with testing made collaboratively between the treating physician and the patient. Those classified as Receptive received their embryo at the same time point as their mock cycle biopsy (*P* + 5 in HRT, or seven days post‐hCG in mNC). Those classified as Pre‐receptive had their transfer delayed by 24 h relative to the biopsy timing, whereas those classified as Post‐receptive had their transfer advanced by 24 h. In cases where two embryos were selected for transfer, both were replaced on the same scheduled transfer day. The second embryo in both the pET and npET groups was a morphologically lower‐grade blastocyst.

**FIGURE 1:**
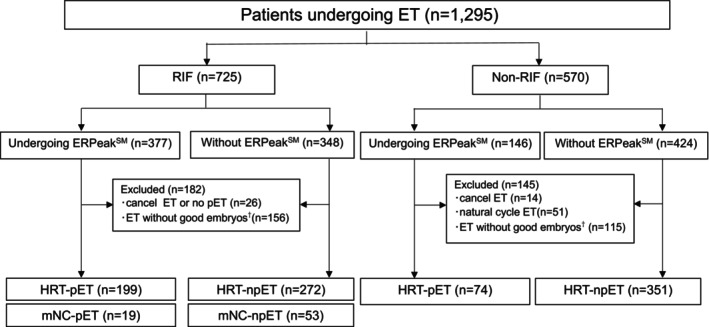
Flowchart of patient selection and study design. Patients undergoing embryo transfer (ET) were divided into recurrent implantation failure (RIF) and non‐RIF groups. They were further categorized based on whether they underwent the ERPeak^SM^ test. Patients who underwent the test received personalized ET (pET), while those who did not received non‐personalized ET (npET). Exclusions included canceled ET, natural cycle ET (for the main analysis), and ET without good‐quality embryos. HRT, hormone replacement therapy; mNC, modified natural cycle.

The following exclusion criteria were applied (Figure 1): patients who opted for a mNC protocol instead of HRT for the primary analysis (mNC patients were retained exclusively for the sub‐analysis), those who did not produce blastocysts of sufficient quality for transfer (defined as a blastocyst graded ≥ 3BB according to the Gardner scoring criteria), those who withdrew from embryo transfer, those who declined pET and chose standard transfer timing, and those whose prior WOI assessment had been performed using a different testing platform.

The primary clinical outcomes—live birth rate (LBR) and miscarriage rate (MR)—were compared between the pET and npET groups, with a prespecified sub‐analysis restricted to euploid embryo transfers. LBR was defined as the proportion of transfers resulting in a live birth beyond 22 completed weeks of gestation. MR was calculated per clinical pregnancy and defined as the proportion of clinically confirmed pregnancies that ended in spontaneous abortion before 13 weeks of gestation. The clinical pregnancy rate (CPR) was defined as the number of pregnancies confirmed by transvaginal ultrasonography showing a gestational sac, divided by the total number of embryo transfer cycles performed. Pregnancies that resulted only in a positive serum *β*‐hCG without ultrasound confirmation (biochemical pregnancies) and ectopic pregnancies were not included in this calculation.

### 2.6 Statistical Analysis

Categorical outcome variables were compared using Fisher's exact test or the Pearson chi‐squared test, as appropriate, while continuous baseline characteristics were assessed with the Mann–Whitney *U* test. Statistical significance was set at a two‐sided *p* value of less than 0.05. Both unadjusted (crude) and adjusted odds ratios (ORs) with corresponding 95% confidence intervals (CIs) were reported. Unadjusted ORs were derived from univariate logistic regression models.

Propensity score matching (PSM) was used to adjust for potential differences in characteristics between the pET and npET groups using multiple logistic modeling. Propensity scoring was performed using a 1:1 nearest‐neighbor matching algorithm with a caliper width of 0.2 standard deviations of the logit of the propensity score. Covariates included maternal age at ovum retrieval, paternal age at ovum retrieval, anti‐Mullerian hormone (AMH) levels, maternal body mass index (BMI), gravidity, parity, duration of infertility, number of previous embryo transfer cycles, and number of embryos transferred. Standardized mean differences (SMD) were used to assess covariate balance, with SMD < 0.1 indicating good balance. EZR software version 4.1.1 (Saitama Medical Center, Jichi Medical University, Saitama, Japan) was used for statistical analysis.

To evaluate the temporal stability of the WOI, we performed a time‐to‐event analysis using the Kaplan–Meier method on patients who underwent repeat ERPeak^SM^ testing (*n* = 117). The “event” was defined as any shift in the WOI status between tests. The median stability time was determined with 95% confidence intervals. Patients were stratified by age (≥ 38 years and < 38 years) and compared using the log‐rank test. Short‐term WOI stability (≤ 12 months) was compared between age groups using Fisher's exact test. Survival analyses were performed using Python 3.11 with the lifelines package.

For the mNC‐FET sub‐analysis, inverse probability of treatment weighting (IPTW) was employed to adjust for potential confounding factors. Propensity scores were estimated using a multivariable logistic regression model with female age, AMH, BMI, and the number of prior embryo transfer cycles as covariates. Stabilized weights were calculated, and covariate balance was assessed using SMD (< 0.10). Weighted logistic regression models were used to compare clinical outcomes. These analyses were performed using Python with the statsmodels package.

## 3 Results

### 3.1 Patient Characteristics

A total of 1295 patients underwent embryo transfer during the study period. After applying the exclusion criteria (Figure 1), 471 RIF patients and 425 non‐RIF patients were included in the primary analysis. In the RIF cohort, 199 patients underwent pET guided by ERPeak^SM^, while 272 received npET. In the non‐RIF cohort, 74 patients underwent pET, and 351 received npET.

Baseline characteristics of the RIF cohort before PSM are summarized in Table 1. The pET group had significantly lower gravidity rate (67.3% vs. 94.9%, *p* < 0.001), shorter infertility duration (54.4 vs. 64.5 months, *p* < 0.001), and slightly more transferred embryos per cycle (1.42 vs. 1.32, *p* = 0.049). Other characteristics, including maternal age, AMH, and BMI, were comparable between groups. After 1:1 PSM, 161 matched pairs were generated, and all baseline covariates achieved excellent balance (standardized mean difference < 0.1 for all variables; Table 2). Baseline characteristics of the non‐RIF cohort before PSM are summarized in Table 3. Compared to the npET group, the pET group was significantly older (37.7 ± 4.7 vs. 35.6 ± 4.3 years, *p* < 0.001), had lower AMH levels (3.00 ± 2.8 vs. 4.57 ± 3.9 ng/mL, *p* < 0.001), more previous failed embryo transfers (1.46 ± 0.7 vs. 0.92 ± 0.8, *p* < 0.001), and more embryos transferred per cycle (1.27 ± 0.5 vs. 1.05 ± 0.2, *p* < 0.001). After 1:1 PSM, 67 matched pairs were generated, and all baseline covariates achieved excellent balance (standardized mean difference < 0.1 for all variables; Table 4).

**TABLE 1:** RIF patients' profiles and reproductive outcomes before Propensity Score Matching of the Personalized Embryo Transfer (pET) and non‐personalized embryo transfer (npET) groups.

	pET	npET	Crude Odds Ratio (95% CI)	*p*‐value
Patients: N	199	272	—	—
Maternal Age (years)	38.1 ± 4.6	38.0 ± 4.3	—	0.83
Paternal Age (years)	41.8 ± 6.5	42.5 ± 6.1		0.24
AMH (ng/mL)	2.17 (1.10–4.44)	2.30 (1.21–4.34)	—	0.41
BMI (kg/m^2^)	20.9 ± 3.5	21.2 ± 2.7	—	0.31
Gravida: N (%)	134 (67.3)	258 (94.9)	—	< 0.001
Parity: N (%)	32 (16.1)	57 (21.0)	—	0.19
Infertility periods (months)	54.4 ± 28.3	64.5 ± 32.7	—	< 0.001
No. of previous failed ET	5.80 ± 2.7	5.50 ± 2.6	—	0.23
No. of transferred embryos per ET	1.42 ± 0.6	1.32 ± 0.5	—	0.049
Clinical pregnancy rate: *N* (%)	106/199 (53.3)	93/272 (34.2)	2.19 (1.51–3.19)	< 0.001
Miscarriage rate: *N* (%)	24/106 (22.6)	32/93 (34.4)	0.56 (0.30–1.04)	0.067
Live birth rate: *N* (%)	82/199 (41.2)	61/272 (22.4)	2.42 (1.62–3.62)	< 0.001

*Note: p*‐values were calculated using Welch's *t*‐test for continuous variables and Fisher's exact test for categorical variables. Data are presented as mean ± SD or median and interquartile range (IQR).

Abbreviations: RIF, recurrent implantation failure; SD, standard deviation.

**TABLE 2:** RIF patient outcomes in the personalized embryo transfer (pET) and non‐personalized embryo transfer (npET) groups after propensity score matching.

	pET	npET	Adjusted Odds Ratio (95% CI)	*p*‐value
Propensity‐matched patients: *N*	161	161	—	—
Maternal Age (years)	38.2 ± 4.5	38.1 ± 4.4	—	0.92
Paternal Age (years)	42.2 ± 6.3	42.1 ± 6.0		0.88
AMH (ng/mL)	2.10 (0.99–4.01)	2.09 (1.05–3.93)	—	0.87
BMI (kg/m^2^)	21.2 ± 3.5	21.1 ± 2.5	—	0.92
Gravida: *N* (%)	125 (77.6)	130 (80.7)	—	0.32
Parity: *N* (%)	27 (16.8)	24 (14.9)	—	0.90
Infertility periods (months)	59.8 ± 27.4	59.4 ± 30.5	—	0.91
No. of previous failed ET	5.95 ± 2.8	5.86 ± 2.9	—	0.77
No. of transferred embryos per ET	1.32 ± 0.6	1.37 ± 0.6	—	0.37
Clinical pregnancy rate of: *N* (%)	87/161 (54.0)	53/161 (32.9)	2.40 (1.52–3.76)	< 0.001
Miscarriage rate: *N* (%)	12/69 (17.4)	25/69 (36.2)	0.37 (0.17–0.82)	0.014
Live birth rate: *N* (%)	66/161 (41.0)	38/161 (23.6)	2.25 (1.39–3.64)	< 0.001

*Note:* Data are presented as mean ± SD or median and interquartile range (IQR). *p*‐values were calculated using Welch's *t*‐test for continuous variables and Fisher's exact test for categorical variables.

Abbreviations: RIF, recurrent implantation failure; SD, standard deviation.

**TABLE 3:** Non‐RIF patients' profiles and reproductive outcomes before propensity score matching of the personalized embryo transfer (pET) and non‐personalized embryo transfer (npET) groups.

	pET	npET	Crude Odds Ratio (95% CI)	*p*‐value
Patients: N	74	351	—	—
Maternal Age (years)	37.7 ± 4.7	35.6 ± 4.3	—	< 0.001
Paternal Age (years)	41.6 ± 6.2	39.8 ± 6.0		0.012
AMH (ng/mL)	2.01 (0.88–4.11)	3.55 (2.00–5.87)	—	< 0.001
BMI (kg/m^2^)	20.8 ± 3.2	20.9 ± 2.6	—	0.75
Gravida: N (%)	29 (39.2)	114 (32.5)	—	0.25
Parity: N (%)	10 (13.5)	40 (11.4)	—	0.57
Infertility periods (months)	39.8 ± 26.7	43.9 ± 28.7	—	0.23
No. of previous failed ET	1.46 ± 0.7	0.92 ± 0.8	—	< 0.001
No. of transferred embryos per ET	1.27 ± 0.5	1.05 ± 0.2	—	< 0.001
Clinical pregnancy rate: N (%)	35/74 (47.3)	174/351 (49.6)	0.91 (0.55–1.51)	0.80
Miscarriage rate: N (%)	10/35 (28.6)	44/174 (25.3)	1.18 (0.53–2.65)	0.68
Live birth rate: N (%)	25/74 (33.7)	130/351 (37.0)	0.87 (0.51–1.47)	0.69

*Note:* Data are presented as mean ± SD or median and interquartile range (IQR). *p*‐values were calculated using Welch's *t*‐test for continuous variables and Fisher's exact test for categorical variables.

Abbreviations: RIF, recurrent implantation failure; SD, standard deviation.

**TABLE 4:** Non‐RIF patient outcomes in the personalized embryo transfer (pET) and non‐personalized embryo transfer (npET) groups after propensity score matching.

	pET	npET	Adjusted Odds Ratio (95% CI)	*p*‐value
Propensity‐matched patients: N	67	67	—	—
Maternal Age (years)	37.7 ± 4.0	37.6 ± 4.9	—	0.95
Paternal Age (years)	40.2 ± 5.4	41.3 ± 6.5		0.27
AMH (ng/mL)	2.45 (0.92–4.28)	2.29 (1.34–3.42)	—	0.85
BMI (kg/m^2^)	20.5 ± 2.2	20.7 ± 3.2	—	0.69
Gravida: N (%)	26 (38.8)	22 (32.8)	—	0.35
Parity: N (%)	9 (13.4)	7 (10.4)	—	0.95
Infertility periods (months)	39.8 ± 26.4	39.9 ± 24.5	—	0.98
No. of previous failed ET	1.46 ± 0.8	1.46 ± 0.7	—	1.00
No. of transferred embryos per ET	1.32 ± 0.6	1.37 ± 0.6	—	1.00
Clinical pregnancy rate of: N (%)	31/67 (46.3)	24/67 (35.8)	1.54 (0.77–3.08)	0.22
Miscarriage rate: N (%)	7/31 (22.6)	7/24 (29.2)	0.71 (0.21–2.39)	0.54
Live birth rate: N (%)	24/67 (35.8)	17/67 (25.4)	1.54 (0.73–3.24)	0.26

*Note: p*‐values were calculated using Welch's *t*‐test for continuous variables and Fisher's exact test for categorical variables. Data are presented as mean ± SD or median and interquartile range (IQR).

Abbreviations: RIF, recurrent implantation failure; SD, standard deviation.

### 3.2 ERPeak^SM^ Test Results

Of 725 RIF patients (mean age: 39.7 ± 4.9 years), 377 (52.0%) underwent receptivity testing and 348 (48.0%) did not (Figure 1). Within this RIF group, ERPeak^SM^ showed 182 (48.3%) non‐receptive (NR) results and 195 (51.7%) receptive (R) results. Of the patients with a displaced window, 87.9% (160/182) indicated a Pre‐receptive state and 12.1% (22/182) showed a Post‐receptive state. Of 570 non‐RIF patients, 146 (25.6%) underwent receptivity testing and 424 (74.4%) did not (Figure 1). Among the 146 non‐RIF patients who underwent testing, 78 (53.4%) patients were found to be Receptive, and 68 patients (46.6%) displayed a displaced window. Of the patients with a displaced window, 83.8% (57/68) indicated a Pre‐receptive state and 16.2% (11/68) showed a Post‐receptive state. There was no significant difference in the prevalence of a displaced WOI between RIF (48.3%) and non‐RIF (46.6%) patients (*p* = 0.82). No patients required a second biopsy, and no cases experienced complications of the test.

### 3.3 Clinical Outcomes in RIF and Non‐RIF Patients

**FIGURE 2:**
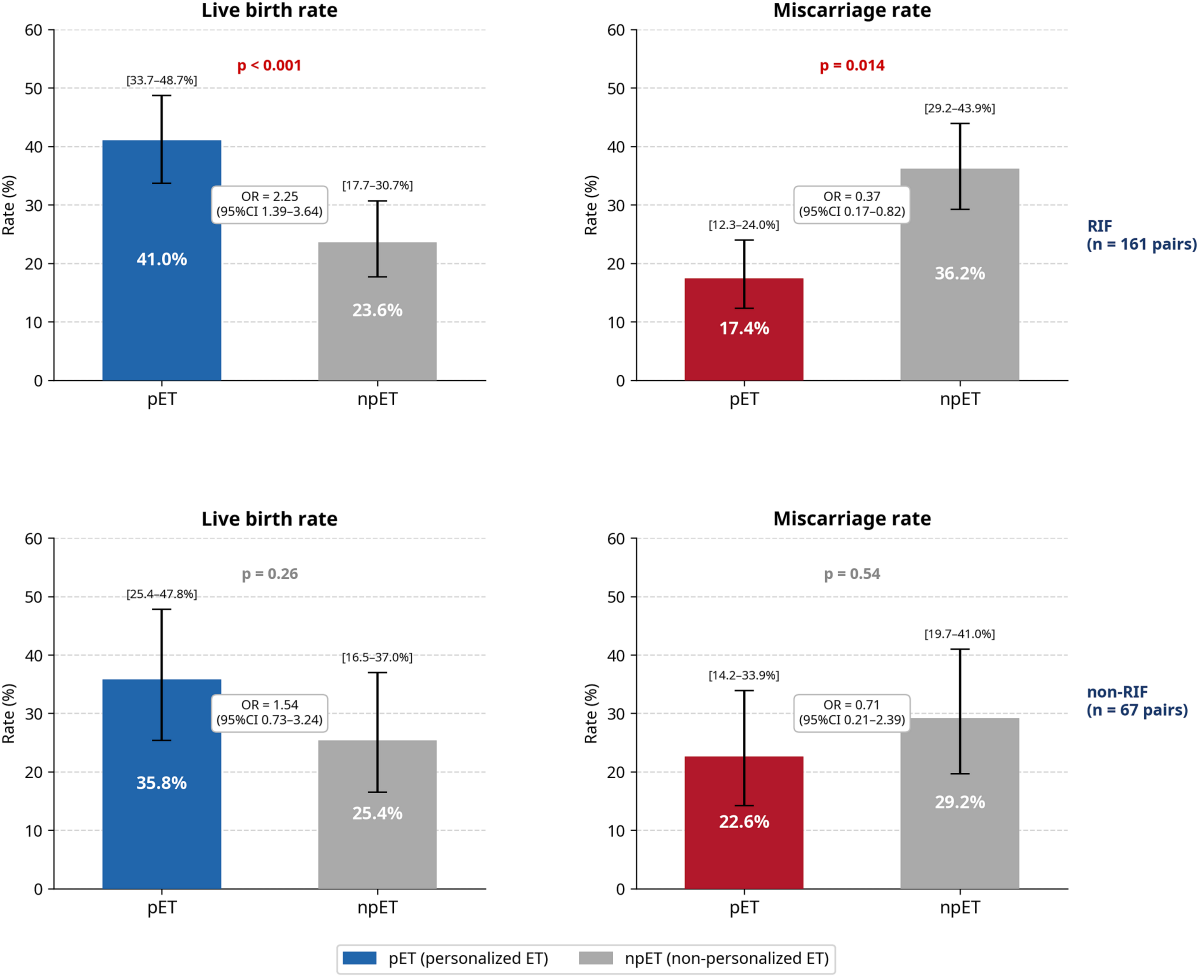
Reproductive outcomes of HRT‐FET after propensity score matching. Comparison of live birth rates (left panels) and miscarriage rates (right panels) between personalized embryo transfer (pET) and non‐personalized embryo transfer (npET) in hormone replacement therapy (HRT) cycles. The upper panels show the outcomes for the recurrent implantation failure (RIF) group (*n* = 161 pairs), and the lower panels show the outcomes for the non‐RIF group (*n* = 67 pairs). Propensity score matching was performed to adjust for baseline characteristics. *p*‐values were calculated using the chi‐squared test or Fisher's exact test. CI, confidence interval; OR, odds ratio.

In the PSM‐adjusted RIF cohort (Table 2), the pET group demonstrated significantly higher LBR (41.0% vs. 23.6%, adjusted OR: 2.25; 95% CI, 1.39–3.64, *p* < 0.001) compared to the npET group. Furthermore, the MR was significantly lower in the pET group (17.4% vs. 36.2%, adjusted OR: 0.37; 95% CI, 0.17–0.82, *p* = 0.014) (Figure 2). When analyzing the unadjusted outcomes based on ERPeak^SM^ test results (Table S1), both Receptive and Non‐receptive pET groups showed significantly higher clinical pregnancy rates and live birth rates compared to the npET group (Receptive: LBR 42.5% vs. 22.4%, OR = 2.55, *p* < 0.001; Non‐receptive: LBR 39.8% vs. 22.4%, OR = 2.29, *p* = 0.002). Interestingly, the Receptive pET group also showed significantly improved outcomes compared to the npET group. In a pre‐specified subgroup analysis of euploid embryo transfers (Table S2), the benefit of pET remained significant in RIF patients (LBR: 54.5% vs. 24.2%, *p* < 0.05), further supporting the clinical utility of individualized timing.

In contrast, in the PSM‐adjusted non‐RIF cohort (*n* = 67 pairs), there were no significant differences between the pET and npET groups in LBR (35.8% vs. 25.4%, adjusted OR: 1.54; 95% CI, 0.73–3.24, *p* = 0.26) and MR (22.6% vs. 29.2%, adjusted OR: 0.71; 95% CI, 0.21–2.39, *p* = 0.54) (Table 4, Figure 2).

### 3.4 IPTW‐Adjusted Outcomes in Modified Natural Cycle FET

A subanalysis was conducted on 72 RIF patients undergoing mNC‐FET with good‐quality embryos (ERPeak^SM^ group, *n* = 19; Control group, *n* = 53). Before adjustment, the ERPeak^SM^ group had higher AMH levels and a slightly higher BMI. After applying stabilized IPTW, excellent covariate balance was achieved across all variables (SMD < 0.10) (Figure S1).

**FIGURE 3:**
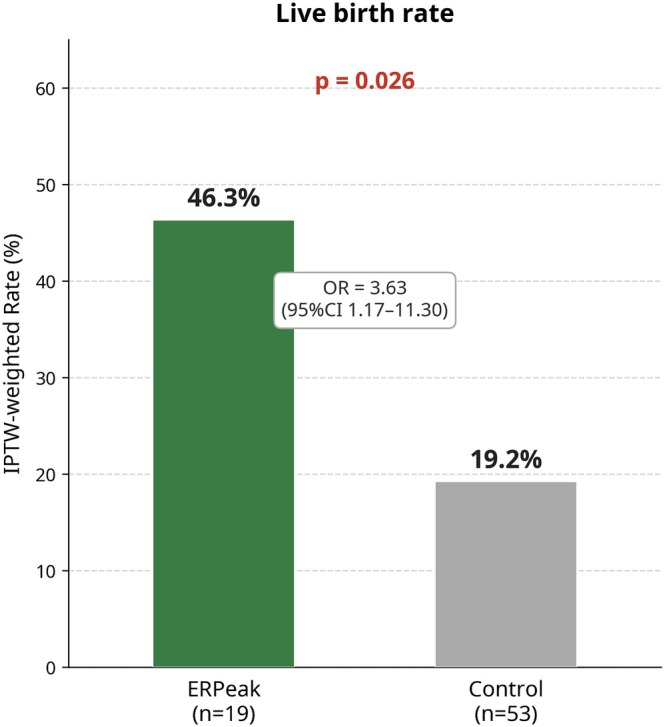
Live birth rate of mNC‐FET after inverse probability of treatment weighting. Comparison of the live birth rate between personalized embryo transfer (ERPeak, *n* = 19) and non‐personalized embryo transfer (Control, *n* = 53) in modified natural cycle (mNC) frozen embryo transfer. Inverse probability of treatment weighting (IPTW) was applied to adjust for confounding factors. The pET group showed a significantly higher live birth rate compared to the npET group (*p* = 0.026). CI, confidence interval; OR, odds ratio.

Weighted logistic regression analysis (Figure 3) demonstrated that ERPeak^SM^‐guided mNC‐FET resulted in a significantly higher LBR (46.3% vs. 19.2%, OR: 3.63; 95% CI, 1.17–11.30, *p* = 0.026) compared to conventional mNC‐FET. The MR was 13.3% in the ERPeak^SM^ group and 5.9% in the control group, with no statistically significant difference (IPTW‐adjusted OR: 2.46; 95% CI, 0.43–14.21, *p* = 0.315), likely reflecting the limited statistical power of this sub‐analysis. The significant improvement in LBR remained robust in the sensitivity analysis using trimmed IPTW.

### 3.5 Temporal Stability of the Window of Implantation

To determine the temporal stability of the WOI, we analyzed 117 observations from 105 patients who underwent multiple ERPeak^SM^ tests. The median interval between tests was 24.0 months (range: 6–48 months). A shift in the WOI status occurred in 52 observations (44.4%).

**FIGURE 4:**
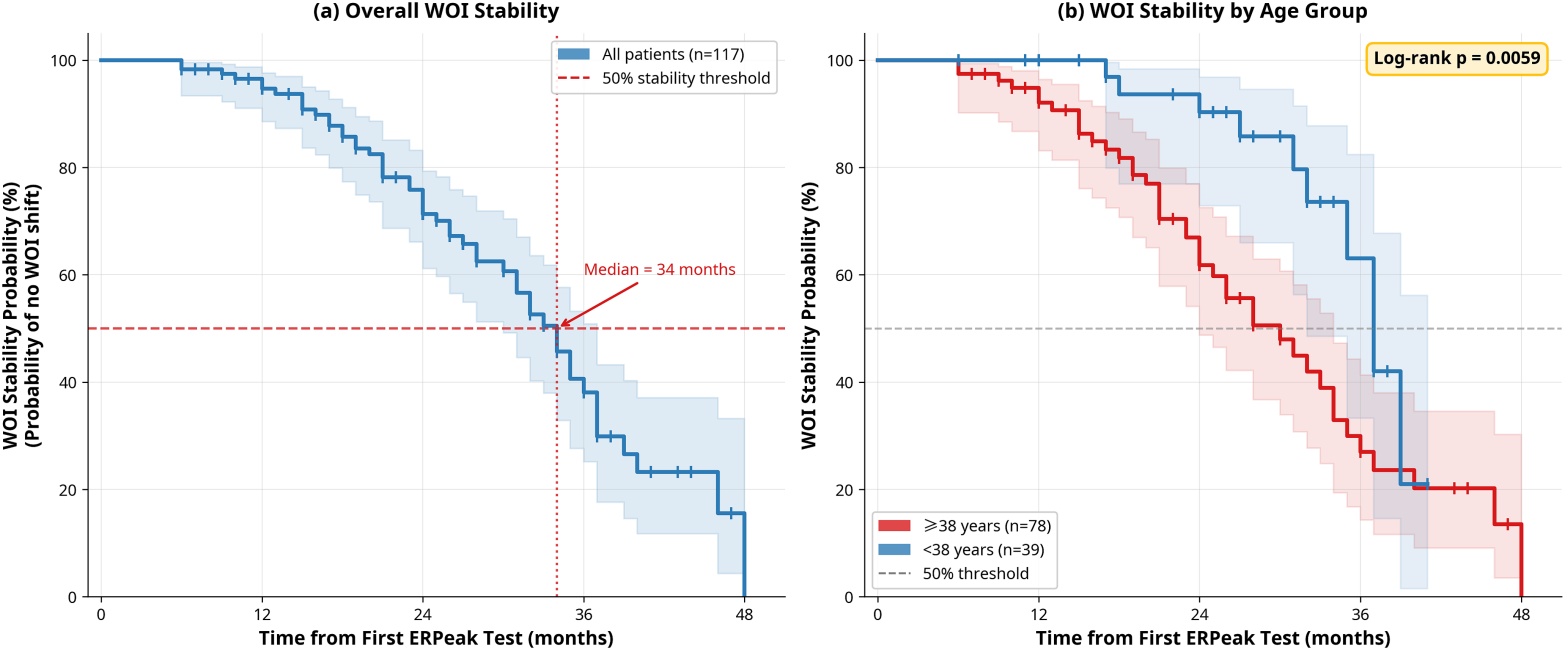
Kaplan–Meier curves for the stability of the window of implantation (WOI). (a) Overall probability of maintaining a stable WOI (no shift) over time from the first ERPeak^SM^ test in all patients (*n* = 117). The median stability duration was 34 months. (b) Comparison of WOI stability between patients aged ≥ 38 years (*n* = 78) and < 38 years (*n* = 39). The older age group showed a significantly faster decline in WOI stability (Log‐rank *p* = 0.0059). Shaded areas represent 95% confidence intervals.

Kaplan–Meier survival analysis (Figure 4) revealed that the overall median time to a WOI shift was 34.0 months. When stratified by age at the time of the first test, patients aged ≥ 38 years (*n* = 78) exhibited a significantly more rapid decline in WOI stability compared to those aged < 38 years (*n* = 39) (log‐rank test, *p* = 0.0059). The median time to a WOI shift was 30.0 months for the older group, whereas the median was not reached in the younger group during the observation period. In short‐term retesting (≤ 12 months), no WOI changes were observed in patients ≤ 37 years (0/7, 0%), whereas 31.3% (5/16) of patients ≥ 38 years showed WOI changes (*p* = 0.130).

## 4 Discussion

This prospective cohort study demonstrates that pET guided by gene expression profiling significantly improves reproductive outcomes in patients with recurrent implantation failure (RIF) but not in non‐RIF patients, providing evidence for targeted rather than universal application of endometrial receptivity testing. Furthermore, our longitudinal analysis reveals that the WOI is a dynamic, physiological state that destabilizes over time, particularly in women of advanced maternal age. Finally, we provide preliminary evidence suggesting that gene‐expression‐guided transfer timing may offer substantial clinical benefits even in mNC‐FET.

Our study demonstrates a distinct difference in the efficacy of pET between RIF and non‐RIF populations. In the PSM‐adjusted RIF cohort, pET significantly improved live birth rates and reduced miscarriage rates, aligning with previous studies demonstrating the utility of endometrial receptivity testing in patients with a history of multiple failed transfers [5, 9]. Conversely, in the non‐RIF cohort, pET provided no significant improvement despite a similar prevalence of displaced WOI. Several factors may explain this discrepancy. First, in non‐RIF patients, a displaced WOI identified by gene expression profiling may not be the primary rate‐limiting step for implantation. Second, non‐RIF patients who chose ERPeak^SM^ testing had multiple adverse prognostic factors (e.g., advanced maternal age, diminished ovarian reserve), suggesting they represented a more challenging subgroup. While PSM adjusted for measured confounders, residual confounding may have obscured any potential benefit. Consequently, our data support the targeted application of ERPeak^SM^ testing specifically for RIF patients, while its routine use in non‐RIF patients is not supported by our data, consistent with recent RCTs showing no benefit of routine testing in unselected populations [10]. Furthermore, since embryonic aneuploidy is the leading cause of implantation failure, confirming euploidy via preimplantation genetic testing for aneuploidies (PGT‐A) before evaluating endometrial receptivity is a highly logical approach. Combining ERPeak^SM^ with PGT‐A may offer a synergistic benefit, maximizing the chance of success for valuable euploid embryos. Regarding the observed reduction in miscarriage rates in the RIF pET group, it is important to note that miscarriage is multifactorial. While our PSM model adjusted for maternal age, AMH, BMI, number of transferred embryos, and prior miscarriage history, and all patients underwent screening for uterine factors, thyroid dysfunction, and thrombophilia, we did not adjust for embryo ploidy status. Therefore, the reduction in miscarriage rates should be interpreted with caution due to these unmeasured confounders.

Interestingly, both Receptive and Non‐receptive pET groups showed significantly improved outcomes compared to the npET group (Table S1). As discussed in our previous report, the benefit observed in Receptive patients may be partly attributable to the endometrial scratching effect of the biopsy procedure itself [5]. Since all patients in the pET group underwent a biopsy in the mock cycle, this mechanical stimulation may have contributed to the improved outcomes observed even in Receptive patients, consistent with reports showing that endometrial scratching improves clinical pregnancy rates in patients with three or more previous implantation failures [18].

Furthermore, our sub‐analysis of mNC‐FET provides preliminary evidence that gene expression‐based WOI testing may benefit select RIF patients even in natural cycles. While mNC‐FET relies on endogenous hormonal fluctuations and is generally considered to have a more physiologic WOI, our IPTW‐adjusted data suggest that a subset of RIF patients may still experience WOI displacement requiring precise synchronization. However, this finding should be interpreted cautiously given the small sample size. Larger prospective studies are needed to confirm these preliminary observations.

A novel and critical aspect of our study is the longitudinal assessment of WOI stability. While the prevailing assumption has been that a patient's WOI remains constant, our Kaplan–Meier analysis revealed that WOI shifted in over 50% of retested cases, with stability being highly age‐dependent. Patients aged ≥ 38 years demonstrated significantly faster WOI changes compared to younger patients. This accelerated loss of WOI stability in older women may be attributed to age‐related physiological changes in the endometrium, including reduced hormone receptor expression [19], cellular senescence [20, 21, 22, 23], and compromised vascularization [24]. From a clinical perspective, these findings provide an evidence base for establishing age‐stratified re‐evaluation guidelines. For patients aged ≥ 38 years, WOI retesting should be considered within 18–24 months of the initial test. Although the median stability (the point at which 50% of patients experience a shift) was 30 months in this age group, waiting for the median is clinically unacceptable when transferring valuable euploid embryos. The recommendation for earlier retesting is driven by the early divergence of the Kaplan–Meier curve and the observation that 31.3% of older patients showed WOI changes even within 12 months. For patients ≤ 37 years, WOI remained completely stable within 12 months, suggesting that routine short‐term retesting may not be necessary. However, re‐evaluation should still be considered after longer intervals or in cases of persistent implantation failure despite a previously receptive result. These recommendations require validation in larger prospective studies with sufficient statistical power to confirm age‐specific thresholds.

This study has several limitations. First, as a single‐center observational study in Japan, our findings on WOI stability are derived from a specific population and may not be generalizable to ethnically and geographically diverse cohorts. The proposed retesting interval serves as a preliminary reference that requires international validation in large‐scale, multicenter randomized controlled trials. Second, selection bias may have influenced our results, as the decision to undergo ERPeak^SM^ testing was not randomized. Although we employed rigorous PSM to adjust for measured confounders, unmeasured variables such as patient compliance and individual variation in progesterone absorption could not be accounted for. Third, the sub‐analysis of mNC‐FET was limited by a relatively small sample size. While stabilized IPTW was used to adjust for confounding variables, wide confidence intervals indicate some uncertainty regarding the exact magnitude of the treatment effect. Finally, regarding the WOI stability analysis, the exact timing of the WOI shift between two consecutive tests cannot be determined (interval censoring), providing a conservative estimate of the retesting interval. Larger prospective studies are needed to definitively establish age‐specific retesting guidelines.

In conclusion, gene expression‐guided personalized embryo transfer is a highly effective intervention for patients with RIF undergoing HRT‐FET. Preliminary evidence also suggests a potential benefit in mNC‐FET, though larger studies are needed. However, its routine use in non‐RIF patients is not supported by our data. Furthermore, clinicians must recognize that the window of implantation is dynamic, and periodic re‐evaluation is crucial, particularly for women of advanced maternal age.

## Ethics Statement

This study was approved by Reproduction Clinic Tokyo Review Board on July 25, 2022 (approval no: RT20722552). All procedures followed were in accordance with the ethical standards of the responsible committee on human experimentation (institutional and national) and with the Helsinki Declaration of 1964 and its later amendments.

## Consent

Informed consent was obtained from all patients for being included in the study.

## Conflicts of Interest

The authors declare no conflicts of interest.

## Supporting information


**Figure S1:** Covariate balance before and after inverse probability of treatment weighting (IPTW) in the modified natural cycle frozen embryo transfer (mNC‐FET) sub‐analysis. Love plot showing standardized mean differences (SMDs) for each covariate before (orange circles) and after (blue diamonds) application of stabilized IPTW. Covariates included the number of prior embryo transfer cycles (prior ET), body mass index (BMI), anti‐Müllerian hormone (AMH) level, and female age at embryo transfer. The red dashed vertical line indicates the SMD = 0.10 threshold. Before IPTW, AMH (SMD = 0.28) and age (SMD = 0.20) exceeded the threshold, indicating baseline imbalance between the ERPeak^SM^ and control groups. After applying stabilized IPTW, all covariates achieved excellent balance, with SMD values falling below 0.10 (prior ET: 0.004; BMI: 0.016; AMH: 0.042; age: 0.016). AMH, anti‐Müllerian hormone; BMI, body mass index; ET, embryo transfer; IPTW, inverse probability of treatment weighting; SMD, standardized mean difference.

**Table S1:** Clinical outcomes stratified by ERPeak^SM^ test result in RIF patients (unadjusted analysis).

**Table S2:** RIF patient outcomes in the personalized embryo transfer (pET) and non‐personalized embryo transfer (npET) groups with euploid embryos after Propensity Score Matching.

## Data Availability

The data that support the findings of this study are available from the corresponding author upon reasonable request.
