# Supplementary figures and images for: Personalized Embryo Transfer Improves Live Birth Rates in Recurrent Implantation Failure: A Propensity Score‐Matched Prospective Cohort Study With Window of Implantation Stability Analysis
Source: Reprod Med Biol. 2026 Jul 22;25(1):e70081. doi: 10.1002/rmb2.70081 (PMC13389642; doi:10.1002/rmb2.70081)

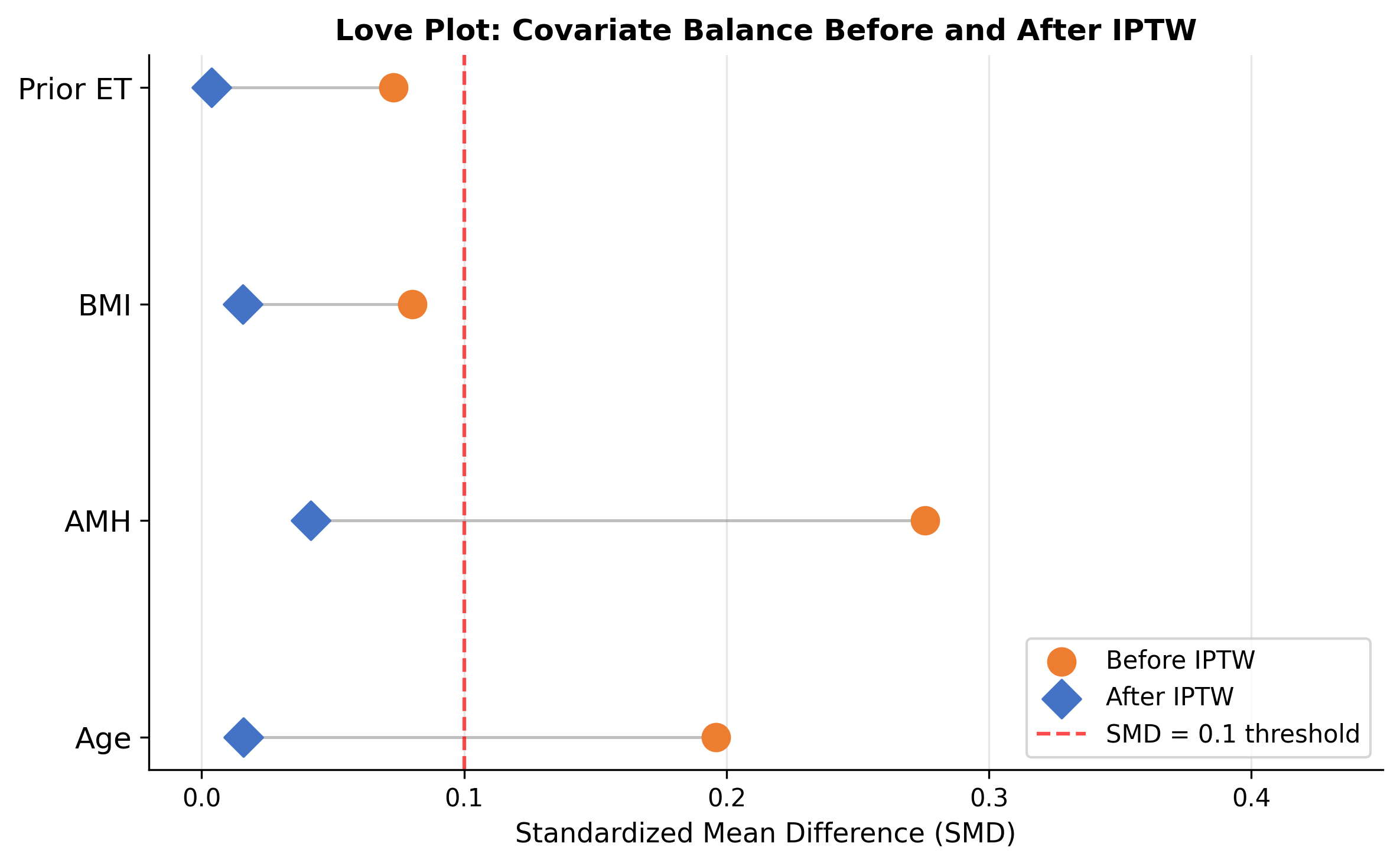

Supplement: Supplementary file 1 — Figure S1: Covariate balance before and after inverse probability of treatment weighting (IPTW) in the modified natural cycle frozen embryo transfer (mNC‐FET) sub‐analysis. Love plot showing standardized mean differences (SMDs) for each covariate before (orange circles) and after (blue diamonds) application of stabilized IPTW. Covariates included the number of prior embryo transfer cycles (prior ET), body mass index (BMI), anti‐Müllerian hormone (AMH) level, and female age at embryo transfer. The red dashed vertical line indicates the SMD = 0.10 threshold. Before IPTW, AMH (SMD = 0.28) and age (SMD = 0.20) exceeded the threshold, indicating baseline imbalance between the ERPeakSM and control groups. After applying stabilized IPTW, all covariates achieved excellent balance, with SMD values falling below 0.10 (prior ET: 0.004; BMI: 0.016; AMH: 0.042; age: 0.016). AMH, anti‐Müllerian hormone; BMI, body mass index; ET, embryo transfer; IPTW, inverse probability of treatment weighting; SMD, standardized mean difference. [file RMB2-25-e70081-s003.png]
