# Supplementary material for: Personalized Embryo Transfer Improves Live Birth Rates in Recurrent Implantation Failure: A Propensity Score‐Matched Prospective Cohort Study With Window of Implantation Stability Analysis
Source: Reprod Med Biol. 2026 Jul 22;25(1):e70081. doi: 10.1002/rmb2.70081 (PMC13389642; doi:10.1002/rmb2.70081)
Supplement: Supplementary file 2 — Table S1: Clinical outcomes stratified by ERPeakSM test result in RIF patients (unadjusted analysis). [file RMB2-25-e70081-s002.docx]

**Table S1. Clinical outcomes stratified by ERPeak^SM^ test result in RIF patients (unadjusted analysis)**

| **Outcome** | **Non-receptive pET (n=93)** | **npET (n=272)** | **OR (95%CI)** | **p-value** | **Receptive pET (n=106)** | **npET (n=272)** | **OR (95%CI)** | **p-value** |
| --- | --- | --- | --- | --- | --- | --- | --- | --- |
| Clinical pregnancy rate | 48/93 (51.6%) | 93/272 (34.2%) | 2.05 (1.27–3.31) | 0.004 | 58/106 (54.7%) | 93/272 (34.2%) | 2.33 (1.47–3.67) | < 0.001 |
| Live birth rate | 37/93 (39.8%) | 61/272 (22.4%) | 2.29 (1.38–3.78) | 0.002 | 45/106 (42.5%) | 61/272 (22.4%) | 2.55 (1.58–4.12) | < 0.001 |
| Miscarriage rate (per clinical pregnancy) | 11/48 (22.9%) | 33/93 (35.5%) | 0.54 (0.24–1.20) | 0.179 | 13/58 (22.4%) | 33/93 (35.5%) | 0.53 (0.25–1.11) | 0.104 |

**Footnote:** Non-receptive: patients classified as Pre-receptive or Post-receptive by ERPeak^SM^ test. Receptive: patients classified as Receptive by ERPeak^SM^ test. npET: non-personalized embryo transfer (patients who did not undergo ERPeak^SM^ testing). OR: odds ratio; CI: confidence interval. p-values were calculated using Fisher's exact test. This analysis is unadjusted; propensity score matching was not applied in this subgroup analysis due to limited sample size in the non-receptive group.
