# Supplementary material for: Personalized Embryo Transfer Improves Live Birth Rates in Recurrent Implantation Failure: A Propensity Score‐Matched Prospective Cohort Study With Window of Implantation Stability Analysis
Source: Reprod Med Biol. 2026 Jul 22;25(1):e70081. doi: 10.1002/rmb2.70081 (PMC13389642; doi:10.1002/rmb2.70081)
Supplement: Supplementary file 3 — Table S2: RIF patient outcomes in the personalized embryo transfer (pET) and non‐personalized embryo transfer (npET) groups with euploid embryos after Propensity Score Matching. [file RMB2-25-e70081-s001.docx]

**Table S2. RIF patient outcomes in the personalized embryo transfer (pET) and non-personalized embryo transfer (npET) groups with euploid embryos after Propensity Score Matching**

| **Variable** | **pET (n=33)** | **npET (n=33)** | **p-value** |
| --- | --- | --- | --- |
| **Section A: Primary outcome – Live Birth Rate (LBR)** |  |  |  |
| **Baseline characteristics (after PSM)** |  |  |  |
| Age (years) | 39.0 (38.0–42.0) | 40.0 (37.0–42.0) | 0.615 |
| AMH (ng/mL) | 2.2 (1.5–5.8) | 2.1 (1.3–5.4) | 0.812 |
| BMI (kg/m²) | 20.8 (19.5–22.2) | 21.3 (20.0–23.4) | 0.359 |
| Prior ET cycles | 7.0 (4.0–9.0) | 6.0 (4.0–8.0) | 0.627 |
| **Outcome** |  |  |  |
| **Live birth rate** | **18/33 (54.5%)** | **8/33 (24.2%)** | **0.023** |
| *Adjusted OR (95% CI)* | *3.75 (1.31–10.7)* | *Reference* | **p < 0.05** |
| **Section B: Primary outcome – Miscarriage Rate (MR)** |  |  |  |
|  | **pET (n=18)** | **npET (n=18)** | **p-value** |
| **Baseline characteristics (after PSM)** |  |  |  |
| Age (years) | 39.0 (35.0–42.8) | 38.5 (35.2–40.0) | 0.484 |
| AMH (ng/mL) | 2.2 (1.3–5.5) | 2.9 (1.9–3.3) | 0.776 |
| BMI (kg/m²) | 20.0 (18.9–22.6) | 20.8 (19.5–21.3) | 0.849 |
| Prior miscarriages | 1.0 (0.0–2.0) | 1.0 (0.2–2.0) | 0.987 |
| **Outcome** |  |  |  |
| **Miscarriage rate** | **3/18 (16.7%)** | **5/18 (27.8%)** | **0.691** |
| *Adjusted OR (95% CI)* | *0.52 (0.10–2.6)* | *Reference* | **p = 0.43** |

*Data are presented as median (interquartile range) for continuous variables and as n/N (%) for categorical variables. p-values for continuous variables were calculated using the Mann–Whitney U test; p-values for categorical variables were calculated using Fisher's exact test. Adjusted odds ratios (ORs) and 95% confidence intervals (CIs) were derived from propensity score-matched logistic regression. Section A: PSM performed with LBR as the primary outcome (n = 33 per group); Section B: PSM performed with MR as the primary outcome (n = 18 per group). AMH, anti-Müllerian hormone; BMI, body mass index; CI, confidence interval; ET, embryo transfer; LBR, live birth rate; MR, miscarriage rate; npET, non-personalized embryo transfer; OR, odds ratio; pET, personalized embryo transfer; PSM, propensity score matching; RIF, recurrent implantation failure.*
